# 5-Benzoyl-4-(4-fluoro­phen­yl)-3,4-dihydro­pyrimidin-2(1*H*)-one

**DOI:** 10.1107/S1600536812052105

**Published:** 2013-01-09

**Authors:** Rajni Kant, Vivek K. Gupta, Kamini Kapoor, D. R. Patil, Madhukar B. Deshmukh

**Affiliations:** a X-ray Crystallography Laboratory, Post-Graduate Department of Physics & Electronics, University of Jammu, Jammu Tawi 180 006, India; b Department of Chemistry, Shivaji University, Kolhapur 416 004(MS), India

## Abstract

In the title mol­ecule, C_17_H_13_FN_2_O_2_, the 3,4-dihydro­pyrimidine ring adopts a flattened sofa conformation with the flap atom (which bears the fluoro­phenyl substituent) deviating from the plane defined by the remaining five ring atoms by 0.281 (2) Å. This plane forms dihedral angles of 85.98 (6) and 60.63 (6)° with the 4-fluoro­phenyl and benzoyl-phenyl rings, respectively. The dihedral angle between the 4-fluoro­phenyl group and the benzene ring is 71.78 (6)°. In the crystal, N—H⋯O hydrogen bonds link mol­ecules into inversion dimers that are further connected by another N—H⋯O inter­action into a two-dimensional supra­molecular structure parallel to (101).

## Related literature

For general background to and pharmaceutical applications of pyrimidino­nes, see: Ghorab *et al.* (2000 ▶); Shivarama Holla *et al.* (2004 ▶); Stefani *et al.* (2006 ▶). For related structures, see: Fun *et al.* (2009 ▶); Chitra *et al.* (2009 ▶). For asymmetry parameters, see: Duax & Norton (1975 ▶).
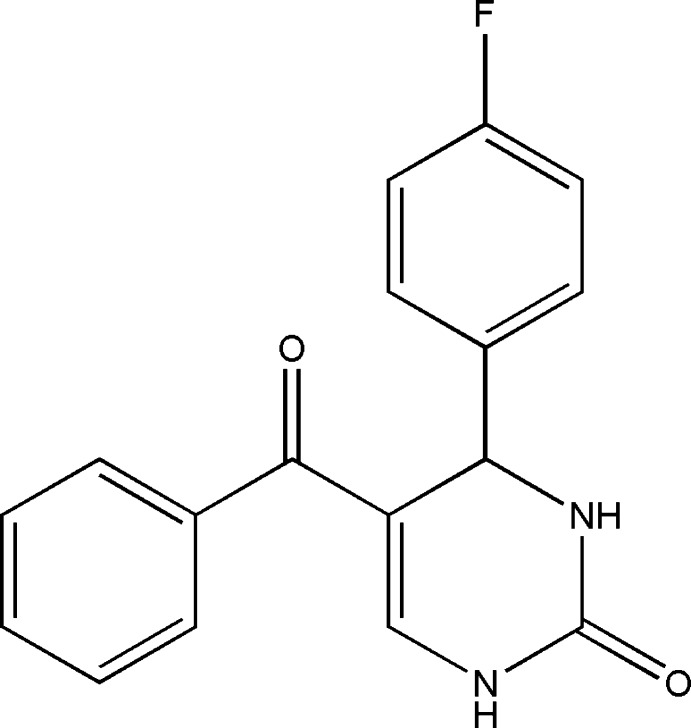


## Experimental

### 

#### Crystal data

C_17_H_13_FN_2_O_2_
*M* *_r_* = 296.29
Monoclinic, 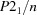

*a* = 12.7911 (5) Å
*b* = 8.1862 (3) Å
*c* = 13.7325 (5) Å
β = 98.850 (4)°
*V* = 1420.82 (9) Å^3^
*Z* = 4
Mo *K*α radiation
μ = 0.10 mm^−1^
*T* = 293 K
0.3 × 0.2 × 0.2 mm

#### Data collection

Oxford Diffraction Xcalibur Sapphire3 diffractometer
Absorption correction: multi-scan (*CrysAlis PRO*; Oxford Diffraction, 2010 ▶) *T*_min_ = 0.777, *T*_max_ = 1.000
27552 measured reflections
2786 independent reflections
1836 reflections with *I* > 2σ(*I*)
*R*_int_ = 0.075

#### Refinement

*R*[*F* ^2^ > 2σ(*F* ^2^)] = 0.048
*wR*(*F* ^2^) = 0.131
*S* = 1.04
2786 reflections
199 parameters
H-atom parameters constrained
Δρ_max_ = 0.21 e Å^−3^
Δρ_min_ = −0.14 e Å^−3^

### 

Data collection: *CrysAlis PRO* (Oxford Diffraction, 2010 ▶); cell refinement: *CrysAlis PRO*; data reduction: *CrysAlis RED* (Oxford Diffraction, 2010 ▶); program(s) used to solve structure: *SHELXS97* (Sheldrick, 2008 ▶); program(s) used to refine structure: *SHELXL97* (Sheldrick, 2008 ▶); molecular graphics: *ORTEP-3 for Windows* (Farrugia, 2012 ▶); software used to prepare material for publication: *PLATON* (Spek, 2009 ▶).

## Supplementary Material

Crystal structure: contains datablock(s) I, global. DOI: 10.1107/S1600536812052105/gk2549sup1.cif

Structure factors: contains datablock(s) I. DOI: 10.1107/S1600536812052105/gk2549Isup2.hkl

Supplementary material file. DOI: 10.1107/S1600536812052105/gk2549Isup3.cml

Additional supplementary materials:  crystallographic information; 3D view; checkCIF report

## Figures and Tables

**Table 1:** Hydrogen-bond geometry (Å, °)

*D*—H⋯*A*	*D*—H	H⋯*A*	*D*⋯*A*	*D*—H⋯*A*
N1—H1⋯O2^i^	0.86	1.96	2.777 (2)	159
N3—H3⋯O1^ii^	0.86	2.12	2.937 (2)	159

Symmetry codes: (i) 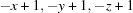; (ii) 
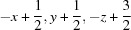
.

**Fig. 1.**
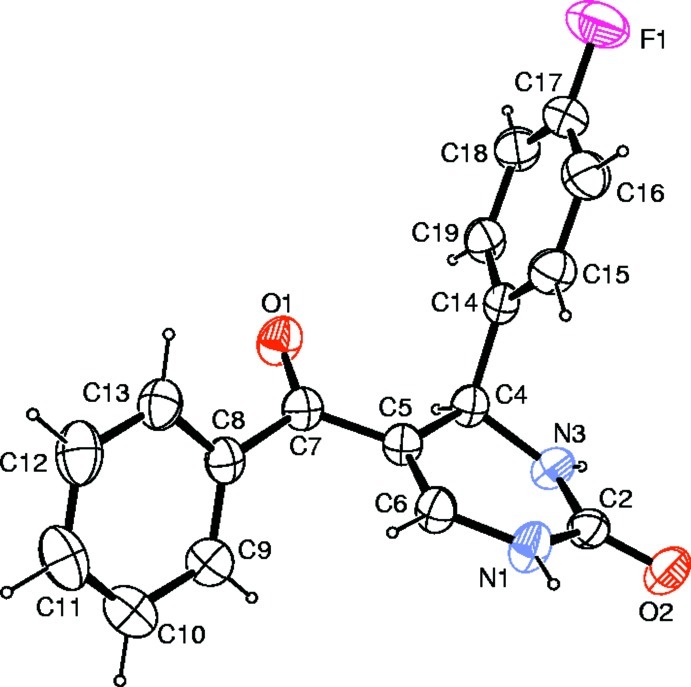
The molecular structure of the title compound with displacement ellipsoids drawn at the 40% probability level. H atoms are shown as small spheres of arbitrary radii.

**Fig. 2.**
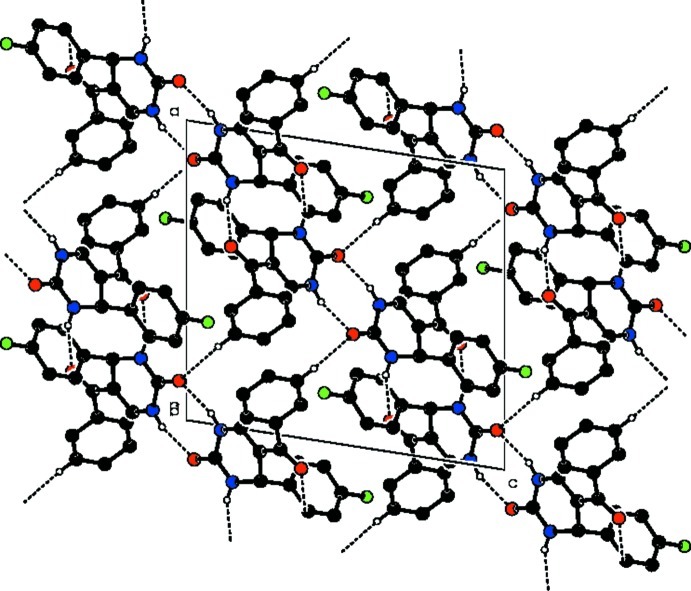
The packing arrangement of molecules viewed along the b axis. The dotted lines show intermolecular N—H···O hydrogen bonds.

## supplementary crystallographic information

### Comment

Dihydropyrimidinones exhibit a wide range of biological effects including antifungal, antiviral, anticancer, antibacterial, anti-inflammatory and antihypertensive (Ghorab *et al.*, 2000; Shivarama Holla *et al.*, 2004). Dihydropyrimidin-2(1H)-ones can also be used as antioxidant agents (Stefani *et al.*, 2006). This paper reports the crystal structure of the title dihydropyrimidinone derivative.

In the title compound (Fig.1) all bond lengths and angles are normal and correspond to those observed in related structures (Fun *et al.*, 2009; Chitra *et al.*, 2009). The dihydropyrimidine ring adopts a sofa conformation (ΔC_s_(C4) = 5.436)(Duax & Norton, 1975) and the plane of the five essentially coplanar atoms (C5/C6/N1/C2/N3) of this ring (maximum deviation -0.045 (2) Å for all atoms) forms a dihedral angle of 85.98 (6)° and 60.63 (6)° with fluorophenyl and benzene ring respectively. In the crystal, N1—H1···O2 hydrogen bonds link molecules into dimers that are further connected by N3—H3···O1 and (Table 1) interactions into two dimensional supramolecular structure (Fig. 2).

### Experimental

A mixture of 3-(dimethylamino)-1-phenylprop-2-en-1-one (1mmol), 4-fluorobenzaldehyde (1mmol), urea (1.2 mmol) and PTSA (30 mol%) in 5 ml ethanol was stirred at 78 °C till the completion of the reaction monitored by TLC. Then reaction mixture was gradually cooled down to room temperature. The precipitate was filtered and washed with cold ethanol (m.p.: 555-557 K, yield: 81%). IR(KBr): 3268, 2964,1682, 1641 ,1592, 1371, 1200, 1151 cm^-1^;^ 1^H NMR(300 MHz, DMSO-d6): δ = 5.45-5.46(d,1H,CH);7.03-7.14(m,3H,Ar-H); 7.35-7.50(m,6H,Ar-H);7.86-7.87(d,1H,NH); 8.21 ( s,1H,CH ); 9.38 ( s,1H,NH );

### Refinement

All H atoms were positioned geometrically and were treated as riding on their parent atoms, with C—H distances of 0.93–0.98 Å and N—H distances of 0.86 Å with *U*_iso_(H) = 1.2*U*_eq_(C/N).

### Crystal data

**Table:**

C_17_H_13_FN_2_O_2_	*F*(000) = 616
*M**_r_* = 296.29	*D*_x_ = 1.385 Mg m^−^^3^
Monoclinic, *P*2_1_/*n*	Mo *K*α radiation, λ = 0.71073 Å
Hall symbol: -P 2yn	Cell parameters from 9924 reflections
*a* = 12.7911 (5) Å	θ = 3.4–29.0°
*b* = 8.1862 (3) Å	µ = 0.10 mm^−^^1^
*c* = 13.7325 (5) Å	*T* = 293 K
β = 98.850 (4)°	Block, colourless
*V* = 1420.82 (9) Å^3^	0.3 × 0.2 × 0.2 mm
*Z* = 4	

### Data collection

**Table:**

Oxford Diffraction Xcalibur Sapphire3 diffractometer	2786 independent reflections
Radiation source: fine-focus sealed tube	1836 reflections with *I* > 2σ(*I*)
Graphite monochromator	*R*_int_ = 0.075
Detector resolution: 16.1049 pixels mm^-1^	θ_max_ = 26.0°, θ_min_ = 3.4°
ω scans	*h* = −15→15
Absorption correction: multi-scan (*CrysAlis PRO*; Oxford Diffraction, 2010)	*k* = −10→10
*T*_min_ = 0.777, *T*_max_ = 1.000	*l* = −16→16
27552 measured reflections	

### Refinement

**Table:**

Refinement on *F*^2^	Primary atom site location: structure-invariant direct methods
Least-squares matrix: full	Secondary atom site location: difference Fourier map
*R*[*F*^2^ > 2σ(*F*^2^)] = 0.048	Hydrogen site location: inferred from neighbouring sites
*wR*(*F*^2^) = 0.131	H-atom parameters constrained
*S* = 1.04	*w* = 1/[σ^2^(*F*_o_^2^) + (0.0593*P*)^2^ + 0.117*P*] where *P* = (*F*_o_^2^ + 2*F*_c_^2^)/3
2786 reflections	(Δ/σ)_max_ = 0.001
199 parameters	Δρ_max_ = 0.21 e Å^−^^3^
0 restraints	Δρ_min_ = −0.14 e Å^−^^3^

### Special details

**Table:**

Experimental. CrysAlisPro, Oxford Diffraction Ltd., Version 1.171.34.40 Empirical absorption correction using spherical harmonics, implemented in SCALE3 ABSPACK scaling algorithm.
Geometry. All esds (except the esd in the dihedral angle between two l.s. planes) are estimated using the full covariance matrix. The cell esds are taken into account individually in the estimation of esds in distances, angles and torsion angles; correlations between esds in cell parameters are only used when they are defined by crystal symmetry. An approximate (isotropic) treatment of cell esds is used for estimating esds involving l.s. planes.
Refinement. Refinement of F^2^ against ALL reflections. The weighted R-factor wR and goodness of fit S are based on F^2^, conventional R-factors R are based on F, with F set to zero for negative F^2^. The threshold expression of F^2^ > 2sigma(F^2^) is used only for calculating R-factors(gt) etc. and is not relevant to the choice of reflections for refinement. R-factors based on F^2^ are statistically about twice as large as those based on F, and R- factors based on ALL data will be even larger.

### Fractional atomic coordinates and isotropic or equivalent isotropic displacement parameters (Å^2^)

**Table:**

	*x*	*y*	*z*	*U*_iso_*/*U*_eq_	
O1	0.39760 (11)	0.04963 (17)	0.86034 (11)	0.0532 (4)	
O2	0.37270 (12)	0.57074 (18)	0.52393 (11)	0.0584 (5)	
F1	0.33786 (14)	0.71433 (18)	1.06679 (11)	0.0932 (5)	
N1	0.48454 (13)	0.3865 (2)	0.60718 (13)	0.0497 (5)	
H1	0.5352	0.4189	0.5774	0.060*	
C2	0.38882 (16)	0.4658 (2)	0.58943 (15)	0.0428 (5)	
N3	0.31734 (13)	0.42049 (19)	0.64458 (12)	0.0436 (4)	
H3	0.2539	0.4556	0.6267	0.052*	
C4	0.33496 (15)	0.3167 (2)	0.73290 (14)	0.0386 (5)	
H4	0.2746	0.2422	0.7299	0.046*	
C5	0.43279 (15)	0.2146 (2)	0.72997 (14)	0.0383 (5)	
C6	0.50143 (16)	0.2583 (2)	0.67049 (15)	0.0429 (5)	
H6	0.5635	0.1983	0.6725	0.051*	
C7	0.45121 (15)	0.0738 (2)	0.79487 (15)	0.0401 (5)	
C8	0.53687 (15)	−0.0452 (2)	0.78012 (15)	0.0406 (5)	
C9	0.54036 (17)	−0.1174 (2)	0.68944 (16)	0.0506 (6)	
H9	0.4915	−0.0867	0.6352	0.061*	
C10	0.61561 (19)	−0.2345 (3)	0.67854 (19)	0.0603 (6)	
H10	0.6164	−0.2836	0.6176	0.072*	
C11	0.6889 (2)	−0.2778 (3)	0.7576 (2)	0.0674 (7)	
H11	0.7398	−0.3562	0.7503	0.081*	
C12	0.68740 (19)	−0.2060 (3)	0.8473 (2)	0.0642 (7)	
H12	0.7382	−0.2348	0.9005	0.077*	
C13	0.61117 (18)	−0.0912 (2)	0.85984 (17)	0.0502 (6)	
H13	0.6098	−0.0450	0.9215	0.060*	
C14	0.33820 (15)	0.4208 (2)	0.82489 (14)	0.0374 (5)	
C15	0.41687 (18)	0.5364 (3)	0.85035 (16)	0.0507 (6)	
H15	0.4704	0.5474	0.8120	0.061*	
C16	0.4170 (2)	0.6356 (3)	0.93181 (17)	0.0598 (6)	
H16	0.4698	0.7131	0.9485	0.072*	
C17	0.3384 (2)	0.6174 (3)	0.98687 (17)	0.0578 (6)	
C18	0.2588 (2)	0.5067 (3)	0.96424 (17)	0.0582 (6)	
H18	0.2049	0.4986	1.0024	0.070*	
C19	0.26005 (17)	0.4065 (2)	0.88326 (16)	0.0480 (5)	
H19	0.2073	0.3283	0.8680	0.058*	

### Atomic displacement parameters (Å^2^)

**Table:**

	*U*^11^	*U*^22^	*U*^33^	*U*^12^	*U*^13^	*U*^23^
O1	0.0473 (9)	0.0574 (9)	0.0592 (9)	0.0000 (7)	0.0219 (8)	0.0136 (7)
O2	0.0593 (10)	0.0675 (10)	0.0529 (9)	0.0202 (8)	0.0223 (8)	0.0227 (8)
F1	0.1272 (14)	0.0846 (10)	0.0730 (10)	−0.0053 (10)	0.0323 (10)	−0.0299 (8)
N1	0.0388 (10)	0.0546 (10)	0.0600 (11)	0.0094 (8)	0.0215 (9)	0.0197 (9)
C2	0.0419 (12)	0.0473 (12)	0.0407 (11)	0.0065 (9)	0.0113 (9)	0.0021 (10)
N3	0.0329 (9)	0.0572 (10)	0.0415 (9)	0.0100 (8)	0.0084 (8)	0.0092 (8)
C4	0.0318 (11)	0.0403 (10)	0.0454 (11)	0.0005 (8)	0.0114 (9)	0.0059 (9)
C5	0.0333 (11)	0.0390 (10)	0.0434 (11)	0.0007 (8)	0.0085 (9)	0.0023 (9)
C6	0.0356 (11)	0.0436 (11)	0.0510 (12)	0.0056 (9)	0.0113 (10)	0.0075 (9)
C7	0.0352 (11)	0.0398 (10)	0.0457 (12)	−0.0046 (9)	0.0076 (9)	0.0013 (9)
C8	0.0356 (11)	0.0356 (10)	0.0518 (12)	−0.0036 (9)	0.0104 (9)	0.0043 (9)
C9	0.0443 (13)	0.0534 (13)	0.0539 (14)	−0.0003 (10)	0.0068 (11)	−0.0008 (10)
C10	0.0544 (15)	0.0568 (14)	0.0713 (17)	0.0000 (12)	0.0145 (13)	−0.0135 (12)
C11	0.0524 (16)	0.0509 (14)	0.099 (2)	0.0110 (11)	0.0109 (15)	−0.0053 (14)
C12	0.0471 (15)	0.0578 (14)	0.0825 (18)	0.0111 (12)	−0.0063 (13)	0.0104 (13)
C13	0.0499 (14)	0.0440 (12)	0.0548 (13)	−0.0032 (10)	0.0022 (11)	0.0048 (10)
C14	0.0361 (11)	0.0353 (10)	0.0418 (11)	0.0028 (9)	0.0089 (9)	0.0067 (8)
C15	0.0477 (14)	0.0549 (13)	0.0522 (13)	−0.0060 (10)	0.0160 (11)	0.0003 (10)
C16	0.0679 (17)	0.0523 (13)	0.0589 (14)	−0.0110 (12)	0.0091 (13)	−0.0048 (12)
C17	0.0790 (18)	0.0484 (13)	0.0480 (13)	0.0049 (12)	0.0159 (12)	−0.0062 (11)
C18	0.0688 (17)	0.0569 (14)	0.0566 (14)	0.0052 (12)	0.0346 (13)	0.0029 (12)
C19	0.0468 (13)	0.0436 (12)	0.0571 (13)	−0.0019 (9)	0.0190 (11)	0.0030 (10)

### Geometric parameters (Å, º)

**Table:**

O1—C7	1.228 (2)	C9—H9	0.9300
O2—C2	1.238 (2)	C10—C11	1.367 (3)
F1—C17	1.355 (2)	C10—H10	0.9300
N1—C6	1.359 (2)	C11—C12	1.369 (3)
N1—C2	1.374 (3)	C11—H11	0.9300
N1—H1	0.8600	C12—C13	1.384 (3)
C2—N3	1.327 (2)	C12—H12	0.9300
N3—C4	1.470 (2)	C13—H13	0.9300
N3—H3	0.8600	C14—C19	1.379 (3)
C4—C5	1.511 (3)	C14—C15	1.387 (3)
C4—C14	1.519 (3)	C15—C16	1.382 (3)
C4—H4	0.9800	C15—H15	0.9300
C5—C6	1.337 (3)	C16—C17	1.356 (3)
C5—C7	1.454 (3)	C16—H16	0.9300
C6—H6	0.9300	C17—C18	1.362 (3)
C7—C8	1.503 (3)	C18—C19	1.384 (3)
C8—C9	1.385 (3)	C18—H18	0.9300
C8—C13	1.387 (3)	C19—H19	0.9300
C9—C10	1.383 (3)		
			
C6—N1—C2	121.89 (17)	C11—C10—C9	119.9 (2)
C6—N1—H1	119.1	C11—C10—H10	120.1
C2—N1—H1	119.1	C9—C10—H10	120.1
O2—C2—N3	123.82 (18)	C10—C11—C12	120.0 (2)
O2—C2—N1	120.08 (18)	C10—C11—H11	120.0
N3—C2—N1	116.09 (17)	C12—C11—H11	120.0
C2—N3—C4	126.95 (16)	C11—C12—C13	120.7 (2)
C2—N3—H3	116.5	C11—C12—H12	119.6
C4—N3—H3	116.5	C13—C12—H12	119.6
N3—C4—C5	108.69 (15)	C12—C13—C8	119.8 (2)
N3—C4—C14	110.09 (14)	C12—C13—H13	120.1
C5—C4—C14	114.58 (16)	C8—C13—H13	120.1
N3—C4—H4	107.7	C19—C14—C15	118.29 (19)
C5—C4—H4	107.7	C19—C14—C4	120.43 (18)
C14—C4—H4	107.7	C15—C14—C4	121.23 (17)
C6—C5—C7	121.81 (17)	C16—C15—C14	121.0 (2)
C6—C5—C4	119.51 (17)	C16—C15—H15	119.5
C7—C5—C4	118.64 (16)	C14—C15—H15	119.5
C5—C6—N1	122.90 (18)	C17—C16—C15	118.6 (2)
C5—C6—H6	118.6	C17—C16—H16	120.7
N1—C6—H6	118.6	C15—C16—H16	120.7
O1—C7—C5	121.36 (17)	F1—C17—C16	118.9 (2)
O1—C7—C8	119.69 (17)	F1—C17—C18	118.5 (2)
C5—C7—C8	118.95 (17)	C16—C17—C18	122.5 (2)
C9—C8—C13	118.75 (19)	C17—C18—C19	118.5 (2)
C9—C8—C7	121.50 (19)	C17—C18—H18	120.7
C13—C8—C7	119.67 (19)	C19—C18—H18	120.7
C10—C9—C8	120.8 (2)	C14—C19—C18	121.0 (2)
C10—C9—H9	119.6	C14—C19—H19	119.5
C8—C9—H9	119.6	C18—C19—H19	119.5
			
C6—N1—C2—O2	−172.51 (19)	C7—C8—C9—C10	−175.79 (18)
C6—N1—C2—N3	6.3 (3)	C8—C9—C10—C11	−1.2 (3)
O2—C2—N3—C4	−169.86 (18)	C9—C10—C11—C12	0.3 (4)
N1—C2—N3—C4	11.4 (3)	C10—C11—C12—C13	1.1 (4)
C2—N3—C4—C5	−22.5 (3)	C11—C12—C13—C8	−1.5 (3)
C2—N3—C4—C14	103.8 (2)	C9—C8—C13—C12	0.6 (3)
N3—C4—C5—C6	17.7 (3)	C7—C8—C13—C12	177.22 (19)
C14—C4—C5—C6	−105.9 (2)	N3—C4—C14—C19	113.42 (19)
N3—C4—C5—C7	−164.33 (16)	C5—C4—C14—C19	−123.7 (2)
C14—C4—C5—C7	72.1 (2)	N3—C4—C14—C15	−64.1 (2)
C7—C5—C6—N1	178.06 (18)	C5—C4—C14—C15	58.8 (2)
C4—C5—C6—N1	−4.0 (3)	C19—C14—C15—C16	−0.3 (3)
C2—N1—C6—C5	−9.6 (3)	C4—C14—C15—C16	177.24 (19)
C6—C5—C7—O1	167.94 (19)	C14—C15—C16—C17	0.1 (3)
C4—C5—C7—O1	−10.0 (3)	C15—C16—C17—F1	−179.9 (2)
C6—C5—C7—C8	−12.9 (3)	C15—C16—C17—C18	−0.8 (4)
C4—C5—C7—C8	169.22 (17)	F1—C17—C18—C19	−179.3 (2)
O1—C7—C8—C9	125.5 (2)	C16—C17—C18—C19	1.6 (4)
C5—C7—C8—C9	−53.7 (2)	C15—C14—C19—C18	1.1 (3)
O1—C7—C8—C13	−51.0 (3)	C4—C14—C19—C18	−176.45 (18)
C5—C7—C8—C13	129.8 (2)	C17—C18—C19—C14	−1.7 (3)
C13—C8—C9—C10	0.7 (3)		

### Hydrogen-bond geometry (Å, º)

**Table:**

*D*—H···*A*	*D*—H	H···*A*	*D*···*A*	*D*—H···*A*
N1—H1···O2^i^	0.86	1.96	2.777 (2)	159
N3—H3···O1^ii^	0.86	2.12	2.937 (2)	159

Symmetry codes: (i) −*x*+1, −*y*+1, −*z*+1; (ii) −*x*+1/2, *y*+1/2, −*z*+3/2.
